# What is Important in E-health Interventions for Stroke Rehabilitation? A Survey Study among Patients, Informal Caregivers and Health Professionals

**DOI:** 10.5195/ijt.2018.6247

**Published:** 2018-08-03

**Authors:** MANON M. WENTINK, LETI VAN BODEGOM-VOS, BERBER BROUNS, HENK J. ARWERT, THEA P.M. VLIET VLIELAND, AREND J. DE KLOET, JORIT J.L. MEESTERS

**Affiliations:** 1 DEPARTMENT OF ORTHOPAEDICS, REHABILITATION MEDICINE AND PHYSICAL THERAPY, LEIDEN UNIVERSITY MEDICAL CENTER, LEIDEN, THE NETHERLANDS; 2 FACULTY OF HEALTH, AMSTERDAM UNIVERSITY FOR APPLIED SCIENCES, AMSTERDAM, THE NETHERLANDS; 3 SOPHIA REHABILITATION CENTRE, THE HAGUE, THE NETHERLANDS; 4 FACULTY OF HEALTH, NUTRITION AND SPORTS, THE HAGUE UNIVERSITY FOR APPLIED SCIENCES, THE HAGUE, THE NETHERLANDS; 5 DEPARTMENT OF BIOMEDICAL DATA SCIENCES, MEDICAL DECISION MAKING, LEIDEN UNIVERSITY MEDICAL CENTER, LEIDEN, THE NETHERLANDS; 6 RIJNLANDS REHABILITATION CENTRE, LEIDEN, THE NETHERLANDS

**Keywords:** Caregivers, E-health, Health professionals, Patients, Rehabilitation, Requirements, Stroke, User-centered design

## Abstract

Incorporating user requirements in the design of e-rehabilitation interventions facilitates their implementation. However, insight into requirements for e-rehabilitation after stroke is lacking. This study investigated which user requirements for stroke e-rehabilitation are important to stroke patients, informal caregivers, and health professionals. The methodology consisted of a survey study amongst stroke patients, informal caregivers, and health professionals (physicians, physical therapists and occupational therapists). The survey consisted of statements about requirements regarding accessibility, usability and content of a comprehensive stroke e-health intervention (4-point Likert scale, 1=unimportant/4=important). The mean with standard deviation was the metric used to determine the importance of requirements. Patients (N=125), informal caregivers (N=43), and health professionals (N=105) completed the survey. The mean score of user requirements regarding accessibility, usability and content for stroke e-rehabilitation was 3.1 for patients, 3.4 for informal caregivers and 3.4 for health professionals. Data showed that a large number of user requirements are important and should be incorporated into the design of stroke e-rehabilitation to facilitate their implementation.

Stroke is a major health problem in Europe for which the incidence is expected to increase from 1.1 million per year in 2000 to 1.5 million per year in 2025 (Truelsen, et al., 2006). Patients suffering from stroke may experience multiple disabilities and require comprehensive rehabilitation. Overall, an increase is expected in the need for rehabilitation post stroke, not only because of the rising incidence, but also since, due to the improvement of the initial medical treatment, more patients now survive a stroke (Feigin et al., 2016). Comprehensive rehabilitation is delivered by various health professionals from different disciplines (e.g., physical therapists, occupational therapists, speech-language pathologists, psychologists, and social workers), with therapy aimed at individual treatment goals involving the patient and his or her informal caregiver (Winstein et al. 2016).

Due to developments in society and health care, including limited resources for the delivery of comprehensive rehabilitation, Information and Communication Technologies (ICT) play an important role in the delivery of rehabilitation care. ‘The use of ICT, mostly internet technology, to improve or support health and health care, is known as e-health (Wentzel, Beerlage-de Jong, & Sieverink, 2014). E-rehabilitation refers to the application of e-health in rehabilitation care (e.g., serious brain games, virtual reality and telerehabilitation). Although many e-rehabilitation interventions have been tested regarding their effectiveness, the use of e-rehabilitation by end users remains low (Brewer, McDowell, & Worthen-Chaudharim, 2007; Lum, Reinkensmeyer, Mahoney, Rymer, & Burgar, 2002).

Implementation of e-health is influenced by its complexity, the adaptability of the technology to fit the local context, and its compatibility with existing systems, work practices, and costs (Ross, Stevenson, Lau, & Murray, 2016). End user input in the design and development of e-health technologies (i.e., user-centered design approach) is a way to overcome such barriers (Goldstein et al., 2014; Pagliari 2007; Ross et al., 2016; van Gemert-Pijnen et al., 2011).

Prior qualitative research (via interviews and focus groups) on end users’ requirements for stroke e-rehabilitation (Ehn et al., 2015; Lange, Flynn, Proffitt, Chang, & Rizzo, 2010; Mawson et al., 2014; Mountain et al., 2006; Nasr et al., 2016; Parker et al., 2014; Zheng et al., 2006) found that interventions should be tailored (Lange et al., 2010; Nasr et al., 2016; Zheng et al., 2006); need to involve goal setting (Mawson et al., 2014; Sivan et al., 2014); must be easy to use; and should provide feedback about training performances (Mawson et al., 2014; Mountain et al., 2006; Nasr et al., 2016; Parker et al. 2014; Zheng et al.,2006).

However, quantitative studies regarding user requirements for e-rehabilitation after stroke are scarce. Thus far, one study used a quantitative survey among 233 health professionals in stroke care to rank the importance of the requirements that were identified in a previous qualitative study of Lu et al., (2011). However, this study was concerned with only one aspect of stroke recovery (upper limb rehabilitation), and one technology tool (robot). Moreover, only health professionals, mainly occupational therapists and physical therapists, completed the survey whereas patients and their informal caregivers were not involved.

Thus, it remains unclear what requirements are most important for the comprehensive delivery of e-rehabilitation interventions (e.g., an app with upper limb exercises, brain games and/or telecommunication) including all potential end users, (i.e., patients, informal caregivers and health professionals). Therefore, this study aims to prioritize the requirements for stroke e-rehabilitation according to patients, informal caregivers, and health professionals. This is relevant for the application of user-centered design and accordingly the development and implementation of effective e-health interventions in stroke rehabilitation.

## PATIENTS AND MATERIALS

### DESIGN AND SETTING

This cross-sectional study, involving a one-time, online survey, was conducted in June 2016 among (former) patients who had been admitted to Sophia Rehabilitation Centre (the Hague) and Rijnlands Rehabilitation Centre (Leiden) in The Netherlands, their informal caregivers, and healthcare professionals (rehabilitation physicians, psychologists, physical therapists and managers). The study was approved by the Medical Ethical Review Board of the Leiden University Medical Center [P15.281].

### STUDY POPULATION

#### PATIENTS AND INFORMAL CAREGIVERS

Patients and informal caregivers were recruited by identifying potentially eligible patients in the electronic patient registries of the two rehabilitation centres, based on the following criteria: older than 18 years, diagnosed with stroke, rehabilitation started after June 2011 and rehabilitation was completed. Four hundred patients (200 in Leiden and 200 in The Hague) were randomly selected by assigning a number to every patient using a random number generator and subsequently selecting the first four-hundred patients and their informal caregivers.

#### HEALTH PROFESSIONALS

Health professionals were selected if they were a practicing health professional (i.e., rehabilitation physicians, physical therapists, or psychologists) with at least two years of working experience in a multidisciplinary team for stroke patients. Health professionals were randomly selected from the Dutch medical address book (which includes most professionals in The Netherlands), the Dutch Association of Rehabilitation Physicians (VRA: Nederlandse Vereniging van Revalidatieartsen) and the Royal Dutch Society of Physical therapy (KNGF: Koninklijk Nederlands Genootschap voor Fysiotherapie). If an email address was missing, other methods (e.g., internet, telephone calls) were used. We aimed to invite at least 300 health professionals.

Patients and health professionals received an email about the study including a digital link to the survey. Informal caregivers (e.g., partner, family member, etc.) were invited to fill in the questionnaire in the email directed to the patients. Thus, it remains unclear whether the patients had an informal caregiver and if so, whether they passed on the invitation. If the invited health professional stated that he or she was not involved in stroke care, they were asked to invite colleagues to fill out the survey. Non-responders received two reminders, each with an in-between period of 1.5 weeks.

### SURVEY DEVELOPMENT

**Figure 1:**
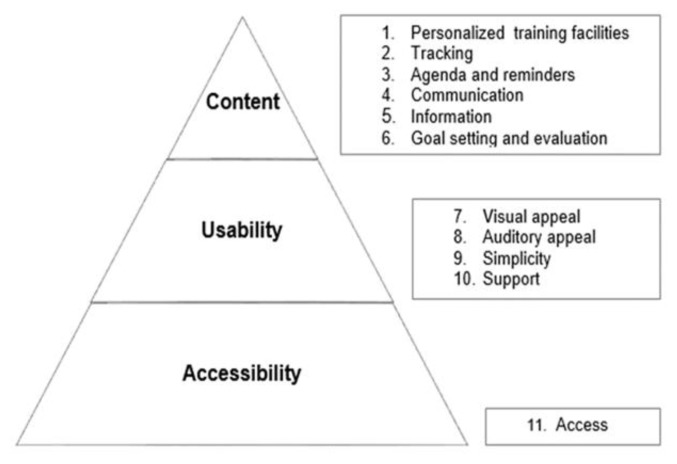
Key themes end-user requirements for e-health interventions in stroke rehabilitation.

The content of the survey was based on a previous qualitative study, in which a framework for end user requirements for e-rehabilitation in stroke care was established (Figure 1). The framework comprises 45 identified requirements, classified into eleven self-determined categories and organized by three self-determined key themes: ‘accessibility’, ‘usability,’ and ‘content’. Accessibility refers to “easy access to e-rehabilitation for all end users, including patients with disabilities as a consequence of stroke.” Usability is “the ease with which end users can use e-rehabilitation interventions for recovery after stroke during their stay in the rehabilitation center and/or at home.” Content was defined as “everything end users want to include in e-rehabilitation (e.g., services, interventions, information, applications, etc.) to achieve specified goals for e-rehabilitation in their rehabilitation process.”

The user requirements identified for patients/ informal caregivers and health professionals were translated into neutral statements for the survey. Each survey consisted of two parts: (1) socio-demographic and disease characteristics, and (2) a list of user requirements for accessibility, usability, and content for patients/ informal caregivers and health professionals. The survey was pilot tested amongst two health professionals and three patients who were undergoing treatment in the rehabilitation center for recovery after stroke. The survey was tested for feasibility, readability and presentation (e.g. perceived question difficulties, response errors, screen layout, etc.). The pilot testing led to minor changes in the wording and format of the final survey.

### SURVEY CONTENT

#### SOCIO-DEMOGRAPHIC (AND DISEASE) CHARACTERISTICS

The age and gender of patients, informal caregivers, and health professionals were recorded. In addition, patients were asked to provide the following information: education level (low [no or only primary education], intermediate [prevocational secondary education, senior secondary vocational training, senior secondary general education, preuniversity education], high [higher professional education or university (bachelor, master, or PhD degree)]); living status (living alone/ living together); employment (paid job/no paid job); time after stroke (in months); and self-perceived impairments as a consequence of stroke (cognitive, physical, communication). Health professionals were asked about their discipline; region (north, middle, and/or south of the Netherlands); work setting (primary care, rehabilitation centre, general hospital); years of work experience; and estimated average number of new stroke patients per month. Moreover, they were asked whether they used e-health in routine stroke rehabilitation (yes, no).

#### USER REQUIREMENTS

Forty-five requirements for the three themes ‘accessibility’ (8 requirements), ‘usability’ (12 requirements) and ‘content’ (25 requirements) of a comprehensive e-health intervention after stroke were identified in the qualitative study and were transformed into neutral statements for the survey. A total of 39/45 requirements were directly transformed and 6/45 requirements were divided into 2 or more statements, resulting in 15 additional statements for the survey (52 statements). The 52 statements were included in the survey for patients. There were 2/52 statements that were accidentally missing in the survey for caregivers, resulting in 50 statements in the survey for caregivers. In the survey of health professionals, a number of 7/52 statements were asked from the perspective of a patient next to their own perspective, resulting in 7 additional statements. There were 11/52 statements derived from the qualitative study were only applicable for patients and caregivers, so eventually 48 statements (52+7–11) were included in the survey of health professionals.

All participants were asked to rate the importance of the given statements on a 4-point Likert scale (1=unimportant, 2=rather unimportant, 3=rather important, 4=important). These scores were used to calculate the mean in order to make a ranking from highest to least important requirements.

### ANALYSIS

Respondents were included in the analyses if they completed ≥90 percent of survey. Socio-demographic and disease characteristics were analyzed using descriptive statistics and presented as numbers with percentages, means with standard deviations (SD), or medians with ranges (Inter Quartile Range; IQR), i.e., 25th percentile–75th percentile), where appropriate.

To quantify the importance of requirements for accessibility, usability and content of e-rehabilitation interventions as perceived by respondents, descriptive analysis was used. The mean with the standard deviation (SD) for each statement were reported to discriminate between and prioritize the statements used in the survey items. Means provide the most accurate insight in the importance of the requirements. Scores on statements per subgroup (patients, informal caregivers and health professionals) are presented in separate tables for each theme: Accessibility, Usability and Content. In addition, the mean score of all statements were provided per subgroup. All statistical analyses were performed using Statistical Packages for the Social Sciences (IBM SPSS 22.0 for Windows).

### ETHICAL ISSUES AND APPROVAL

Participants filled in the survey anonymously implying that patient’s, informal caregiver’s and health professional’s characteristics were not traceable, (e.g., age instead of date of birth). Immediately after filling in the survey, participants were thanked for their willingness to participate. Participants did not receive results of the study, since they filled in the survey anonymously.

## RESULTS

### RESPONSE

**Figure 2:**
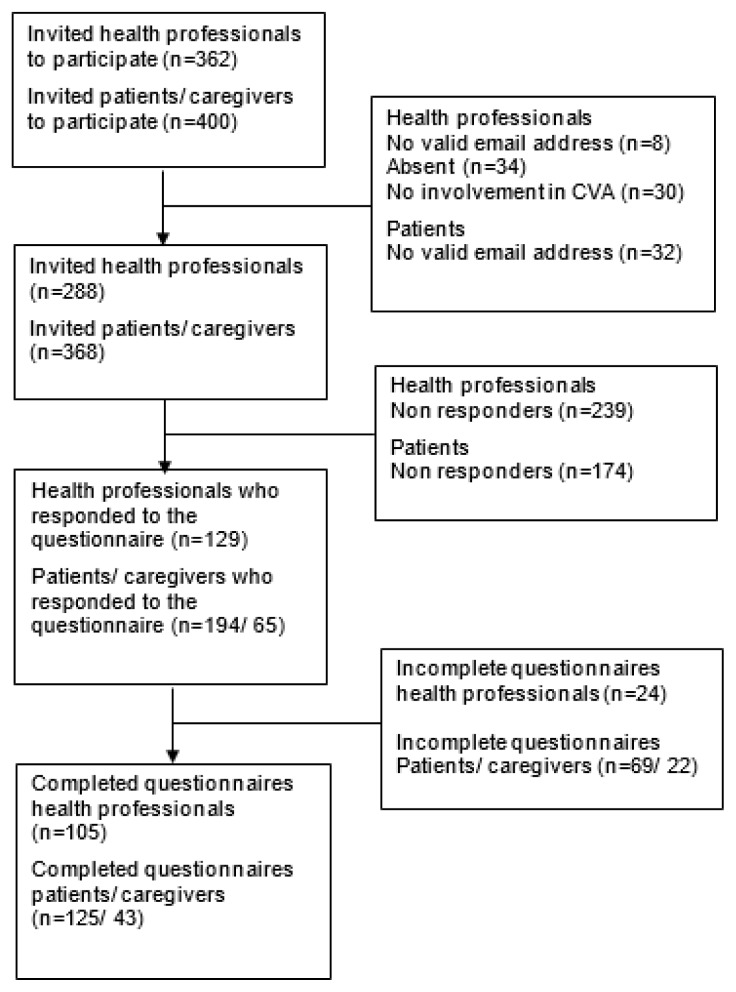
Flow of inclusion.

Of the 400 invited patients, 32 had no valid email address; the survey was completed by 125 out of 368 invited patients (34%). Additionally, 43 informal caregivers, and 105 health professionals completed the survey (Figure 2). Reasons for nonresponse were not verified.

### SOCIO-DEMOGRAPHIC (AND DISEASE) CHARACTERISTICS

The characteristics of the 273 responders are shown in Table 1. Respondents included 72/125 (58%) patients, 16/43 (37%) informal caregivers and 25/105 (24%) health professionals. The mean age of the patients was 58 years (SD 11.4), of the informal caregivers 58 years (SD 12.0) and of the health professionals 42 years (SD 10.5). In total, 41/105 (39%) of the health professionals were physical therapists, 15/105 (14%) were psychologists, 47/105 (45%) were physicians and 2/105 (2%) did not mention their discipline. Seventy-five out of 105 (71%) responding professionals worked in a rehabilitation center.

### REQUIREMENTS

The mean score of all user requirements regarding accessibility, usability and content for stroke e-rehabilitation was 3.1 for patients, 3.4 for informal caregivers and 3.4 for health professionals. For patients, the mean score (SD) for the least important requirement was 2.4 (1.1) and for the most important 3.6 (0.8). For caregivers, the mean score (SD) for the least important requirement was 2.8 (1.1) and for the most important 3.8 (0.4). For health professionals, the mean score (SD) for the least important requirement was 2.4 (1.0) and for the most important 3.9 (0.4).

#### ACCESSIBILITY

Two requirements for accessibility to e-rehabilitation after stroke were found to be the most important according to all end users: *e-rehabilitation is applicable to most commonly possessed ICT-devices, e.g., laptop, tablet and smartphone* (patients: mean 3.5, SD 0.9; informal caregivers: mean 3.5, SD 0.7; professionals: mean 3.6, SD. 0.6) and *access for health professionals to the electronic patient record to stay informed about training results* (patients: mean 3.3, SD 1.0; informal caregivers: mean 3.5, SD 0.9; professionals: mean 3.5, SD 0.7) (see table 2a).

#### USABILITY

Categories for usability were: visual appeal, auditory appeal, simplicity and support. Two requirements regarding the category ‘support’ were found to be most important according to all end users: *videos with instructions on how to use e-rehabilitation* (patients: mean 3.3, SD 1.0; informal caregivers: mean 3.7, SD 0.9; professionals: mean 3.7, SD 0.6) and *a menu with frequently asked questions for patients* (patients: mean 3.1, SD 1.0; informal caregivers: mean 3.7, SD 0.9; professionals: mean 3.7, SD 0.6) (see table 2b).

Moreover, three requirements showed a mean score higher than the mean score on all statements for both patients and informal caregivers: *limited options on a single screen to click further to another screen* within the category simplicity (patients: mean 3.1, SD 1.1; informal caregivers: mean 3.4, SD 1.0)*, non-flashing and tranquil interface* (patients: mean 3.3, SD 0.8; informal caregivers: mean 3.8, SD 0.4) and *adjustable font style and font size settings* (patients: mean 3.0, SD 1.1; informal caregivers: mean 3.6, SD 0.7) within the category visual appeal.

#### CONTENT

Categories for content were: training facilities, tracking, agenda/ reminders, communication, information and goal setting/ evaluation. A relatively large number of requirements for content showed higher mean scores than the mean score on all statements by all end users, e.g., *insight in agreements made during a consult* in the category information (patients: mean 3.5, SD 0.9; informal caregivers: mean 3.6, SD 0.8; professionals: mean 3.7, SD 0.6), *insight in final reports of a patients’ rehabilitation process* in the category information (patients: mean 3.6, SD 0.7; informal caregivers: mean 3.7, SD 0.8; professionals: mean 3.4, SD 0.8) and *physical exercises* in the category training facilities (patients: mean 3.4, SD 1.0; informal caregivers: mean 3.7, SD 0.8, professionals: mean 3.6, SD 0.6).

## DISCUSSION

The aim of this study was to make an inventory and prioritize the requirements for stroke e-rehabilitation according to patients, informal caregivers, and health professionals. Relatively large mean scores for user requirements regarding accessibility, usability and content for a comprehensive e-health intervention after stroke were found for each subgroup (patients 3.1, informal caregivers 3.4 and health professionals 3.4). Moreover, similarities and differences were found between the perspectives of patients, informal caregivers, and health professionals about the importance of the requirements.

To our knowledge one previous study used a quantitative survey in stroke care to discover the importance of the requirements that were identified in a previous qualitative study of Lu et al. (2011). Similar to the findings from the perspective of health professionals in this study, provision of feedback for patient and therapist and the tool being useful for stroke patients were found to beedd important requirements. However, comparison of the findings between both studies is hampered. Lu et al. (2011) focused on the user’ requirements regarding a robot for upper limb rehabilitation, while our study was concerning a comprehensive e-health intervention using multiple tools. Moreover, their survey was conducted among 233 health professionals while our study also included other user groups (i.e., patients and their informal caregivers).

Overall, requirements prioritized in this study were both similar and different compared to previous qualitative studies that identified user requirements for an e-health intervention. An important requirement regarding accessibility found in this study was the ability to use e-rehabilitation on multiple digital devices (smartphone, tablet, laptop). This corresponds to requests identified in previous literature that e-health be integrated in familiar and existing tools/applications, (not replacing them) (Matthew-Maich et al., 2016); is available alongside the work of health professionals (Mountain et al., 2006); is easy to set-up (Sivan et al., 2014; Zheng et al., 2006); and is suitable to the constant modification of the environment (Ross et al., 2016).

An important requirement found in this study regarding usability was a non-flashing and tranquil interface. This is in line with a previous study of Parker et al., (2014) that found participants preferred a simpler looking screen without additional background pictures. In contradiction to previous studies in which design settings needed to be changeable for adjustment to a patient’s needs (Parker et al., 2014; Zheng et al., 2006), this study found changeable lay-out, background and color settings were less important. It can be added to the literature that incorporation of support for use of e-rehabilitation programmes (e.g., helpdesk, FAQ menu, videos with instructions) are highly important. These requirements should be integrated in e-rehabilitation designs to increase acceptance of e-rehabilitation for stroke patients, who often suffer from disabilities impairing usage.

Regarding content, the following important requirements were similar to previous literature: general information (McKevitt, Redfern, Mold, & Wolfe, 2004; Peoples, Satink, & Steultjens, 2011; Reed, Wood, Harrington, & Paterson, 2012; Salter, Hellings, Foley, & Teasell, 2008); goal setting and evaluation (Lu et al., 2011; McKevitt et al., 2004; Parke et al., 2015); and providing feedback (Hochstenbach-Waelen & Seelen, 2012; Lu et al., 2011; Mawson et al., 2015; Mountain et al., 2006; Nasr et al., 2016; Parker et al., 2014; Zheng et al., 2006). In contradiction, telecommunications in stroke care are rapidly developing worldwide because of their importance (Blacquiere et al., 2017), although this was found a less important requirement in the current study according to all end users. A broad range of requirements regarding content of comprehensive e-health interventions can be added to the literature (e.g., exercises for cognitive and physical functioning, hyperlinks to webpages, a reminder function, etc.), since this study prioritized user requirements for a comprehensive e-health intervention instead of a single e-health intervention addressing one aspect of stroke recovery with one technology tool.

Furthermore, similarities were found in perspectives of patients, informal caregivers, and health professionals about the importance of requirements. The requirements of physical exercises, insight in information discussed during a consult, insight in final reports of the rehabilitation process and setting and evaluation of goals for e-rehabilitation were considered relatively important by all end users. However, notable differences were also found between the subgroups. The required exercises for cognitive functioning were important for patients and informal caregivers, whilst this was a less important requirement for health professionals. In addition, health professionals found contact with peers for caregivers and patients important, although patients and informal caregivers found this less important. Moreover, psycho-education was found to be a relatively important requirement by health professionals and informal caregivers, whereas patients seemed to find this less important. Therefore, differences in the importance of user requirements should be identified so that e-health interventions can be designed in such a way that requirements of different users are met.

As to our knowledge, this is the first study that prioritized a set of requirements for e-rehabilitation amongst multiple subgroups (patients, informal caregivers, and health professionals) and in which informal caregivers were treated as a separate group of end users. Differences in the importance of requirements for comprehensive e-health interventions for recovery after stroke between patients, informal caregivers, and professionals were not previously identified in the literature.

A limitation of the study is that selection bias might have occurred since the survey was distributed by mail, and only patients and their informal caregivers with an email address were able to fill in the survey. As a consequence, the perspective of patients and their informal caregivers with least experience with digital devices might be missing. However, we aimed to identify user requirements for e-rehabilitation, so knowledge and understanding of ICT and e-health were desirable. Moreover, informal caregivers of patients were invited to fill in the questionnaire in the invitation mail directed to the patients. If the invited health professional stated that he or she was not involved in stroke care, they were asked to invite colleagues to fill out the survey. Therefore, the actual amount of invited informal caregivers and health professionals and the response rates are unknown.

In summary, we prioritized requirements for accessibility, usability, and content of comprehensive e-health interventions from the perspective of patients, informal caregivers, and health professionals. It was found that a relatively large amount of user requirements were found important by each separate group and by all subgroups. These results can be used by developers, researchers and health professionals to apply user-centered design to develop effective e-health interventions and accordingly to enable their acceptance and adoption in stroke rehabilitation. However, more research is needed to identify which requirements are most important to optimize implementation, usage and adaptation of e-health in stroke rehabilitation.

## Figures and Tables

**Table 1:** Characteristics of Participating Patients, Informal Caregivers and Health Professionals

Characteristics	Patients (n=125)	Caregivers (n=43)	Professionals (n=105)
Age, years (mean, SD)	58 (11.4)	58 (12.0)	42 (10.5)
Sex, no. male (%)	72 (58)	16 (37)	25 (24)
Education, no. (%)			
Low	21 (17)		-
Intermediate	46 (37)		-
High	57 (46)		-
Living status, no. living alone (%)	22 (18)	5 (12)	
Employment, no. with a paid job (%)	42 (34)	21 (49)	
Work region, no. (%)			
North	-	-	20 (20)
Middle	-	-	63 (60)
South	-	-	21 (20)
Health professional discipline, no. (%)			
Physical therapist	-	-	41 (39)
Psychologist	-	-	15 (14)
Physician	-	-	47 (45)
Unknown^a^			2 (2)
Work setting, no. (%)			
Health centre in primary care	-	-	10 (10)
Rehabilitation centre	-	-	75 (71)
General hospital	-	-	34 (32)
Work experience, no. years (%)	-	-	
>0–5	-	-	25 (23.8)
>6–10	-	-	28 (26.7)
>11–15	-	-	14 (13.3)
>15			37 (35.2)
Estimated average number of new stroke patients per month; no. (%)			
>0–5	-	-	47 (46)
>6–10	-	-	33 (32)
>11–15	-	-	11 (11)
>15	-	-	11 (11)
Time after stroke, months (mean, SD)	30.6 (29.2)		
Self-perceived impairments, no. yes (%)			
Cognitive impairments	81 (65)		-
Physical impairments	84 (67)		-
Aphasia	48 (38)		-
Use of any device in daily life, no. yes (%)	113 (90)	41 (95)	
Use of device, no. yes (%)			-
Smartphone	85 (68)	33 (77)	-
Tablet	62 (50)	30 (70)	-
Laptop	71 (57)	30 (70)	-
Computer (PC)	54 (43)	20 (47)	
Use of digital rehabilitation tools, no. yes (%)	-	-	40 (38)

a Health professionals who did not mention their discipline.

**Table 2a:** Importance of Requirements for *Accessibility* of Stroke e-Rehabilitation According to End Users (n=273)

Category	Requirement	End user		
		Patients (n=125) Mean (SD)	Caregivers (n=43) Mean (SD)	Professionals (n=105) Mean (SD)
*The mean score of all statements per subgroup*		*3.3*	*3.5*	*3.4*
Access	Applicable to most commonly possessed ICT-devices.	3.5 (0.9)	3.5 (0.7)	3.6 (0.6)
Access	No internet connection is required to use e-health interventions (offline use).	3.2 (1.0)	3.5 (1.0)	3.1 (0.9)
Access	Different e-health interventions are accessible without logging into the system each time.	3.1 (1.0)	3.3 (0.9)	3.5 (0.8)
Access	Access for health professionals to the electronic patient record to stay informed about training results.	3.3 (1.0)	3.5 (0.9)	3.5 (0.7)

**Table 2b:** Importance of Requirements for *Usability* of Stroke e-Rehabilitation According to End Users (n=273)

Category	Requirement	End user		
		Patients (n=125) *Mean (SD)*	Caregivers (n=43) *Mean (SD)*	Professionals (n=105) *Mean (SD)*
*The mean score of all statements per subgroup*		*2.9*	*3.4*	*3.5*
Visual appeal	Adjustable settings: background.	2.6 (1.1)	3.0 (0.1)	-
Visual appeal	Adjustable settings: colors.	2.5 (1.1)	3.3 (0.1)	-
Visual appeal	Adjustable settings: page lay-out.	2.7 (1.1)	3.3 (0.9)	-
Visual appeal	Adjustable settings: font style and font size.	3.0 (1.1)	3.6 (0.7)	-
Visual appeal	Use of pictograms, symbols and graphics.	2.7 (1.1)	3.3 (1.0)	-
Visual appeal	Non-flashing and tranquil interface.	3.3 (0.8)	3.8 (0.4)	-
Auditory appeal	Ability to listen to written text.	2.7 (1.1)	3.4 (1.0)	-
Auditory appeal	Sounds for alert or as feedback.	2.7 (1.1)	3.3 (0.9)	-
Simplicity	Limited amount of open webpages as a consequence of using a service.	2.8 (1.0)	3.6 (0.9)	3.5 (0.7)
Simplicity	Limited amount of information on a single screen.	3.3 (1.0)	Missing^a^	-
Simplicity	Limited options on a single screen to click further to another screen.	3.1 (1.1)	3.4 (1.0)	-
Support	Direct assistance at home.	3.3 (1.08)	3.1 (1.1)	-
Support	Video for patients with instructions on how to use e-rehabilitation.	3.3 (1.0)	3.7 (0.9)	3.7 (0.6)
Support	Video for professionals with instructions on how to use e-rehabilitation.	-	-	3.1 (0.8)
Support	Menu with frequently asked questions for patients (FAQ).	3.1 (1.0)	3.7 (0.9)	3.7 (0.6)
Support	Menu with frequently asked questions for professionals (FAQ).	-	-	3.3 (0.7)
Support	A helpdesk for patients.	2.9 (1.1)	3.5 (0.9)	3.9 (0.4)
Support	A helpdesk for professionals.	-	-	3.5 (0.6)

a This requirement was accidentally missing in the survey for caregivers.

**Table 2c:** Importance of Requirements for *Content* of Stroke e-Rehabilitation According to End Users (n=273)

Category	Requirement	End user		
		Patients (n=125) *Mean (SD)*	Caregivers (n=43) *Mean (SD)*	Professionals (n=105) *Mean (SD)*
*The mean score of all statements per subgroup*		*3.0*	*3.2*	*3.2*
Training facilities	Exercises for cognitive functioning.	3.6 (0.9)	3.8 (0.4)	3.2 (0.9)
Training facilities	Physical exercises.	3.4 (1.0)	3.7 (0.8)	3.6 (0.6)
Training facilities	Speech exercises.	2.9 (1.3)	3.5 (1.0)	3.3 (1.0)
Tracking	Monitor activities in daily living:			
	Insight in completed activities	2.5 (1.1)	3.1 (1.1)	3.2 (0.8)
	Duration of completed activities	3.1 (1.0)	3.6 (0.7)	3.3 (0.7)
Tracking	A video system to record exercises at home.	2.4 (1.1)	2.8 (1.1)	3.1 (0.9)
Tracking	Monitor a patient’s health status:			
	Body weight	2.9 (1.1)	3.2 (1.0)	2.5 (0.9)
	Heart rate	2.9 (1.1)	Missing^a^	2.5 (0.8)
Agenda/reminders	Insight in the rehabilitation schedule of a patient.	3.2 (1.1)	3.4 (1.0)	3.6 (0.7)
Agenda/reminders	A reminder function for scheduled appointments.	2.9 (1.0)	3.4 (1.0)	3.7 (0.6)
Agenda/reminders	Scheduled time to use e-rehabilitation (digital training).	3.2 (1.1)	3.3 (1.0)	3.3 (0.8)
Agenda/reminders	Appointments with healthcare professionals:			
	Make a request for an appointment.	3.1 (1.0)	3.3 (1.0)	2.9 (0.9)
	Schedule an appointment themselves.	3.0 (1.0)	3.2 (1.0)	2.5 (1.0)
Communication	Contact for caregivers to share experiences on how to cope with having a relative with stroke.	2.6 (1.1)	2.7 (1.0)	3.6 (0.6)
Communication	Contact for patients to share experiences on how to cope with having a stroke.	2.8 (1.0)	3.0 (1.0)	3.5 (0.6)
Communication	Communication between patients, caregivers and professionals from a distance (telecommunication).	2.5 (1.1)	3.0 (1.0)	2.9 (0.9)
Information	General information about stroke.	3.4 (0.8)	3.4 (0.9)	3.7 (0.5)
Information	Hyperlinks to reliable and relevant webpages for patients and caregivers.	3.2 (0.9)	3.4 (0.5)	3.6 (0.5)
Information	Information about patient organizations.	3.3 (1.0)	3.0 (1.0)	3.7 (0.6)
Information	Information on how to cope with consequences of stroke (psycho-education).	2.8 (1.0)	3.7 (0.8)	3.6 (0.6)
Information	Descriptions on how to perform daily activities (strategy training).	2.4 (1.2)	3.1 (0.9)	3.3 (0.7)
Information	Insight after a consult with a health professional in:			
	Agreements that were made	3.5 (0.9)	3.6 (0.8)	3.7 (0.6)
	Information that was discussed	3.5 (0.8)	3.7 (0.7)	3.4 (0.8)
Information	Insight in final reports of a patients’ rehabilitation process.	3.6 (0.7)	3.7 (0.8)	3.4 (0.8)
Goal setting/evaluation	Setting goals for e-rehabilitation (shared decision-making).	3.4 (0.9)	3.7 (0.8)	3.4 (0.7)
Goal setting/evaluation	Evaluation of goals for e-rehabilitation.	3.4 (0.9)	3.7 (0.7)	3.4 (0.7)
Goal setting/evaluation	Feedback about training results for patients:			
	Insight in what is trained	3.2 (1.0)	3.6 (0.6)	2.3 (1.1)
	The number of completed sessions	3.1 (0.9)	3.6 (0.6)	3.5 (0.7)
	Training outcomes	3.2 (1.0)	3.7 (0.5)	3.5 (0.7)
Goal setting/evaluation	Feedback about training results for professionals:			
	Insight in what is trained	-	-	3.2 (0.8)
	The number of completed sessions	-	-	3.2 (0.8)
	Training outcomes	-	-	3.3 (0.8)
Goal setting/evaluation	Feedback on when a goal for e-rehabilitation is accomplished.	3.3 (0.9)	3.6 (0.9)	3.7 (0.6)
Goal setting/evaluation	Use of clinical assessments for goal setting and evaluation.	3.5 (0.9)	3.6 (0.7)	3.3 (0.8)
Goal setting/evaluation	Use of valid questionnaires for goal setting and evaluation.	3.3 (0.9)	3.5 (0.8)	3.4 (0.8)
Goal setting/evaluation	Compare training outcomes of a single patient with those of other patients.	2.4 (1.2)	2.9 (1.0)	2.4 (1.0)

a This requirement was accidentally missing in the survey for caregivers.
